# The Use of a Composite of Modified Construction Aggregate and Activated Carbon for the Treatment of Groundwater Contaminated with Heavy Metals and Chlorides

**DOI:** 10.3390/ma18153437

**Published:** 2025-07-22

**Authors:** Katarzyna Pawluk, Marzena Lendo-Siwicka, Grzegorz Wrzesiński, Sylwia Szymanek, Osazuwa Young Osawaru

**Affiliations:** 1 Department of Hydraulic Engineering, Technology and Works Organisation, Institute of Civil Engineering, Warsaw University of Life Sciences, Nowoursynowska 159, 02-787 Warsaw, Poland; marzena_lendo_siwicka@sggw.edu.pl (M.L.-S.); grzegorz_wrzesinski@sggw.edu.pl (G.W.); sylwia_szymanek@sggw.edu.pl (S.S.); 2 Łukasiewicz Research Network-Industrial Chemistry Institute, Rydygiera 8, 01-793 Warsaw, Poland; osazuwa.osawaru@ichp.lukasiewicz.gov.pl

**Keywords:** composite of construction aggregate and activated carbon, MgO modification, remediation, contaminant mixture

## Abstract

The treatment of contaminants from road infrastructure poses significant challenges due to their variable composition and the high concentrations of chloride ions, heavy metals, and oil-derived substances. Traditional methods for protecting groundwater environments are often insufficient. A promising alternative is permeable reactive barrier (PRB) technology, which utilizes recycled materials and construction waste as reactive components within the treatment zone of the ground. This paper delves into the potential of employing a composite (MIX) consisting of modified construction aggregate (as recycled material) and activated carbon (example of reactive material) to address environmental contamination from a mixture of heavy metals and chloride. The research involved chemical modifications of the road aggregate, activated carbon, and their composite, followed by laboratory tests in glass reactors and non-flow batch tests to evaluate the kinetics and chemical equilibrium of the reactions. The adsorption process was stable and conformed to the pseudo-second-order kinetics and Langmuir, Toth, and Redlich–Peterson isotherm models. Studies using MIX from a heavy metal model solution showed that monolayer adsorption was a key mechanism for removing heavy metals, with strong fits to the Langmuir (R^2^ > 0.80) and Freundlich models, and optimal efficiencies for Cd and Ni (R^2^ > 0.90). The best fit, at Cd, Cu, Ni = 0.96, however, was with the Redlich–Peterson isotherm, indicating a mix of physical and chemical adsorption on heterogeneous surfaces. The Toth model was significant for all analytes, fitting Cl and Cd well and Pb and Zn moderately. The modifications made to the composite significantly enhanced its effectiveness in removing the contaminant mixture. The test results demonstrated an average reduction of chloride by 85%, along with substantial removals of heavy metals: lead (Pb) by 90%, cadmium (Cd) by 86%, nickel (Ni) by 85%, copper (Cu) by 81%, and zinc (Zn) by 79%. Further research should focus on the removal of other contaminants and the optimization of magnesium oxide (MgO) dosage.

## 1. Introduction

The selection of reactive materials for constructing permeable reactive barriers necessitates careful consideration of both the properties of the materials and the nature of the pollutants. Pollutants from road infrastructure present unique challenges due to their heterogeneous composition and seasonal fluctuations in concentrations. Notably, there are periods during which the chloride ion concentrations exceeded the permissible threshold of 1000 mg/L, as stipulated by the 2019 Regulation of the Minister of Maritime Economy and Inland Navigation (Journal of Laws 2019, Item 1311) [1]. Furthermore, elevated concentrations of heavy metals and oil-derived substances are often detected.

Laboratory analyses indicate that existing ground and water protection measures, such as filter screens fabricated from sand or gravel, are insufficient as barriers against these pollutants. Consequently, permeable reactive barrier (PRB) technology, which utilizes recycled materials and construction waste as reactive filling materials, presents a promising alternative. This approach not only reduces installation costs but also supports the circular economy and reduces the amount of waste going to landfills. One of the most commonly used materials is scrap iron, especially in the form of zero-valent iron (ZVI), which effectively reduces many inorganic and organic pollutants, including chlorinated compounds. Metal waste of this type is readily available and has high chemical reactivity [2]. Another example is fly ash, a by-product of coal combustion, which, thanks to its sorption properties, can bind heavy metals and improve the physical structure of the barrier. Among the materials originating from building demolition, such as crushed bricks or concrete, there are components with high porosity that support mechanical filtration and can also participate in sorption processes [3]. Recycled crushed glass, on the other hand, thanks to its durability and chemical neutrality, can act as a filter medium and, in some cases, support chemical reactions in the barrier [3,4]. The utilization of reactive materials in environmental protection, industrial applications, and agriculture is inherently linked to the necessity of continuous enhancements in quality, performance, and production methodologies. The academic literature outlines various techniques for modifying these materials, classified as either physical (thermal) or chemical modifications.

Physical modification involves the application of thermal processes to induce oxidation or reduction reactions on specific functional groups on the surface of the material. This approach is well-documented in multiple studies, including those by Cheng et al. [5], Rongyue et al. [6], Stavropoulos [7], and Wang et al. [8]. Conversely, chemical modification employs oxidizing or reducing agents to generate diverse types of surface functional groups on the materials. The chemical modification of reactive substances, such as activated carbon, zeolite, limestone, and silica, has been the subject of numerous investigations, as documented in the works of Arora et al. [9], Gao et al. [10], Marzaioli et al. [11], Lan et al. [12], and Lin et al. [13].

One notable example of a substance utilized in chemical modifications is magnesium oxide. Magnesium oxide (MgO), often referred to as roasted magnesia, is a non-flammable, white crystalline solid that is obtained through the combustion of magnesium or the roasting of magnesite (magnesium carbonate) or dolomite. This compound is distinguished by its unique morphological and microstructural characteristics, such as small particle size and high homogeneity. It also exhibits excellent insulating properties, mechanical strength, and non-toxicity. As a refractory and corrosion-resistant material, magnesium oxide serves a variety of applications. Thanks to its high surface reactivity, magnesium oxide can function as a catalyst in several processes, including condensation, conversion, and hydrogenation. Additionally, it possesses notable chemical adsorption capabilities. Magnesium oxide is widely employed in the production of cements, castings, fireproof vessels, and crucibles, as well as serving as an inerting agent in medicine and photography [14,15]. Moreover, it has a strong affinity for forming ionic compounds with chlorine [16]. Recent reviews also highlight MgO’s potential for enhancing the performance of cementitious materials [4], as well as its application in water treatment for the remineralization and removal of divalent metals [17].

This paper aims to present the modification of reactive materials to enhance their efficacy in the removal of typical pollutants found in road surface runoff. It further examines the potential application of a mixture comprising modified road aggregate and activated carbon in mitigating environmental impacts arising from a combination of heavy metal and chloride contaminants. The initial hypothesis posits that materials enhanced with magnesium oxide (MgO) may significantly improve the removal of both chloride and metal ions from aqueous solution retention due to increased surface activity and chemical reactivity.

## 2. Materials and Methods

### 2.1. Materials

This study investigated two materials: activated carbon SAR-1 (AC), sourced from the Experimental Plants for the Production of Activated Carbon in Mrozy, Poland, and construction aggregate (CA) material derived from road demolition in the Mazovian Voivodeship. The selection of these materials was driven by the activated carbon’s broad capacity for contaminant removal and its substantial specific surface area, while the road aggregate was chosen due to its availability at no cost.

All batch tests were performed using model solutions prepared with distilled water and specific doses of contaminants. The following analytical-reagent-grade salts from CHEMPUR (Piekary Śląskie, Poland) were used to formulate the model solutions:

- Cadmium chloride dihydrate (CdCl_2_·2.5H_2_O);
- Copper(II) chloride dihydrate (CuCl_2_·2H_2_O);
- Nickel(II) sulphate heptahydrate (NiSO_4_·7H_2_O);
- Lead(II) nitrate (Pb(NO_3_)_2_);
- Zinc chloride (ZnCl_2_);
- Sodium chloride (NaCl).

For the kinetic tests, chloride ions were used with an initial concentration of 150 mg/L. In the equilibrium tests, concentrations of 150, 170, 190, 220, and 250 mg/L were employed. A mixture of heavy metals—cadmium (Cd), copper (Cu), nickel (Ni), lead (Pb), and zinc (Zn)—was also examined, with an initial concentration of each metal set at 60 mg/L for the kinetic tests, along with varying concentrations of 1, 2, 5, 10, 15, and 20 mg/L for equilibrium test. These solutions were prepared in 100 mL Class A volumetric flasks. The appropriate doses of reactants, in the form of salts, were added to 5 mL of distilled water, and the volume was brought up to the mark with additional distilled water.

### 2.2. Materials Modifications

To enhance the capacity of the selected materials for retaining a mixture of contaminants, namely Cl, Cd, Cu, Ni, Pb, and Zn, chemical and thermal modifications were implemented.

The material was treated with a solution of magnesium oxide (MgO), known for its unique morphological and microstructural characteristics, as well as its mechanical strength and non-toxicity. Its high surface reactivity allows MgO to function as a catalyst in various processes, including condensation, conversion, and hydrogenation. Furthermore, it possesses significant chemical adsorption capacity and a strong tendency to form ionic compounds with chlorine [16]. The application of magnesium oxide in the treatment of groundwater media is primarily warranted by its minimal environmental impact and low solubility. This compound functions as a weak base, and its decomposition is an endothermic process that reaches a peak pH of 10. The resulting precipitate is a crystalline substance [18]. Nonetheless, the high cost of magnesium oxide—ranging from 8 to 10 times that of alternative materials—renders its use as a reactive material in PRB economically unfeasible.

For testing purposes, 100 g of individual materials with grain diameters ranging from 1 to 2 millimeters were accurately weighed for each sample and pre-treated in a laboratory oven at a temperature of 105 °C for a duration of 24 h. The modification of the materials was conducted utilizing a 10% magnesium oxide (MgO) solution, which was prepared using distilled water in conjunction with 7 g of magnesium nitrate (V) salt (Mg(NO_3_)_2_·H_2_O). A loading of 10% MgO was selected based on research [19,20], which demonstrates that moderate loadings (approximately 10–12%) facilitate the even dispersion of MgO, optimize adsorption sites, and are cost-effective. In comparison, lower loadings (5%) are insufficient for providing adequate active sites, while higher loadings (30%) may result in an agglomeration and blockage of the pores.

Prior to the modification process, a saturation test was performed on the materials, henceforth referred to as “pure.” For this test, 20 g of construction aggregate and 10 g of activated carbon were uniformly distributed across the surface of a Petri dish to ensure comprehensive coverage by the grains. Subsequently, 0.1 mL portions of distilled water were applied using an automatic pipette until all pores were saturated (when the point was reached at which the added water was no longer absorbed by the sample). The saturation value (N) was found to be 25% for construction aggregate and 125% for activated carbon. The saturated surface dry (SSD) was calculated using the following formula [21]:

(1) $$SSD=\left(\frac{M_{SSD}-M_{DRY}}{M_{DRY}}\right)\times 100\%$$

where MSSD and MDRY are the dry and saturated sample mass, respectively [g].

The volume of the solution employed for treating the materials was established based on their absorbability values. For the construction aggregate sample weighing 100 g, the requisite volume of the solution was determined to be 12.5 mL, whereas for the activated carbon sample of the same weight, the volume was 125 mL.

The prepared materials were modified by uniformly applying the MgO solution in portions with the automatic pipette, followed by drying with cold air. Successive layers of MgO solution were applied at two-hour intervals until the prepared solution was fully utilized and all pores of the modified materials were saturated. Thereafter, the materials were subjected to drying at a temperature of 120 °C for 24 h.

**Figure 1:**
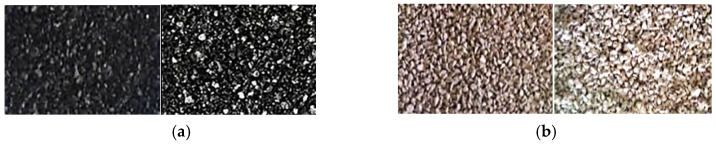
Research materials before and after modifications: (**a**) activated carbon; (**b**) construction aggregate.

Subsequently, the materials underwent thermal modification in a muffle furnace, initially at 250 °C, with a gradual increase to 450 °C. During this thermal processing, magnesium nitrate decomposed into nitric oxide and magnesium oxide, which subsequently crystallized and filled both the pore surfaces and the material surfaces. The nitrogen oxides generated were effectively extracted from the furnace chamber using a vacuum pump. A photographic comparison of the materials before and after modification with 10% MgO is presented in Figure 1.

### 2.3. Preliminary Flow Tests

**Figure 2:**
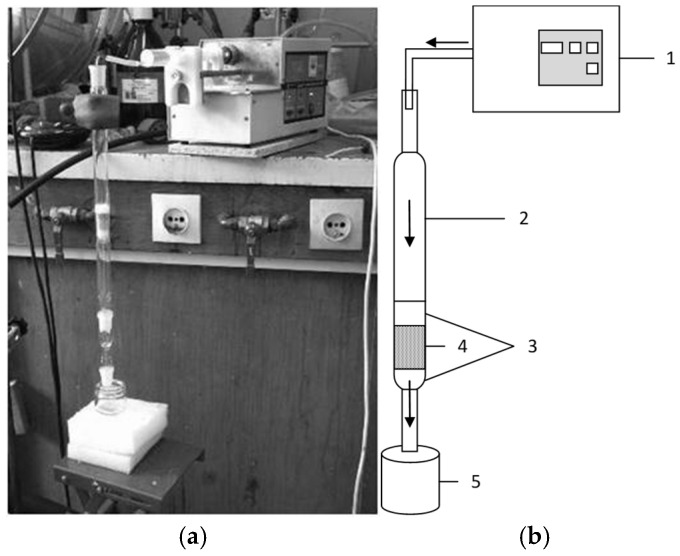
Test bench for preliminary flow tests: (**a**) view; (**b**) scheme, where 1—syringe pump with test solution, 2—glass reactor, 3—glass wool, 4—modified material, 5—filtrate receiver.

Preliminary flow tests were conducted on a bench, as depicted in Figure 2. The first batch of materials modified with 10% MgO included the following weights: construction aggregate MCA—5.0936 g and activated carbon MAC—1.9689 g. For comparison, the weights of the pure materials were construction aggregate CA—6.700 g and activated carbon AC—1.6936 g. These materials were carefully packed using a glass dipstick within a glass reactor, layered between two sections of quartz cotton wool, which had a total mass of 0.2080 g and did not react with the test solution. The quartz wool was employed to prevent the materials from washing out during the flow of the test solution, with the thickness of the test material set at 4 cm. The test solution consisted of a 10% chloride solution, administered from the top to the bottom of the reactor via a syringe pump operating at a flow rate of 1.39 × 10^−7^ m^3^/s. Filtrate was collected at the outlet into a receiver. The objective of this study was to compare the performance of pure and modified materials while evaluating the initial reduction and filtration properties of the modified materials. Prior to and following the test, the weights of the materials, the glass reactor, and the receiver were recorded. The test conditions are summarized in Table 1. The removal ratio R_R_ (%) of chloride by the materials was calculated using the equation:

(2) $$R_{R}=\left(\frac{C_{0}-C}{C_{0}}\right)\times 100\%$$

where c_0_ and c are the initial and final indicator concentrations (mg/L).

### 2.4. Batch Test

Batch studies were conducted using a modified composite (MIX) of activated carbon (AC) and construction aggregate (CA) to explore the reaction kinetics and chemical equilibrium. The results from the reaction kinetics studies provided significant insights into the mechanisms and intensity of the interactions taking place. The removal ratio (R) [%] serves as a basis for determining the duration necessary for the fundamental tests to achieve chemical equilibrium. The removal ratio (R) was calculated using Formula (1).

Numerous mathematical models have been proposed to conceptualize adsorption, which can be broadly categorized into reaction adsorption models and diffusion models. Both categories aim to effectively describe the kinetic adsorption process, albeit with differing foundational approaches. Diffusion models encompass several phenomena, including external or layer diffusion, which refers to the diffusion occurring at the liquid interface surrounding the adsorbent particles; internal or intramolecular diffusion, which pertains to the movement of liquid within pores and along the surfaces of those pores; and mass actions, which involve the processes of adsorption and desorption between the adsorbate and the active sites on the adsorbent [22]. In contrast, the reaction models of chemical reaction kinetics focus on the adsorption process through the lens of a singular phenomenon from the aforementioned categories [23]. This study analyzed the kinetics of the respective reactions through the application of various reaction models, including pseudo-first order, pseudo-second order, Elovich, first order, second order, and the Weber–Morris interpretation of the intramolecular diffusion model [24,25,26].

Additionally, the research aimed to assess the chemical equilibrium of the reactions occurring between the contaminants and the reactive materials, specifically to determine the ability of these materials to retain contaminants during the adsorption process. Given the complexity inherent in adsorption phenomena, a diverse array of models, characterized by varying physical parameters, was employed to interpret the study results. The data were analyzed utilizing several adsorption isotherm models, which included Langmuir, pseudo-Langmuir, anti-Langmuir, Freundlich, Henry, Temkin, Thot, and Redlich–Peterson [27,28]. The adequacy of the models (kinetic and equilibrium) was evaluated by calculating the coefficient of determination (R^2^), using the following formula:

(3) $$R^{2}=1-\frac{\sum_{i=1}^{n}{\left[{{(q}_{e})}_{i\;}{\left(\hat{q_{e}}\right)}_{i}\right]}^{2}}{\sum_{i=1}^{n}{\left[{{(q}_{e})}_{i\;}{\left(\bar{q_{e}}\right)}_{i}\right]}^{2}}$$

where ${{(q}_{e})}_{i}$ represents the measured mass adsorbed per unit mass of the adsorbate [mg/g], ${\left(\hat{q_{e}}\right)}_{i}$ signifies the theoretical mass adsorbed per unit mass of the adsorbate [mg/g], and ${\left(\bar{q_{e}}\right)}_{i}$ is the average measured mass adsorbed on a unit mass of the adsorbate [mg/g].

Additionally, the quality and statistical reliability of the adsorption kinetics and isotherm models were evaluated using the Fisher test (F-test). This test compares the variance explained by the model to the residual variance, helping to determine whether the observed relationship is statistically significant [29]. In all instances, models that exhibited high F-values and low *p*-values (*p* ≤ 0.05) were considered statistically valid. The application of the F-test confirmed that the fitted kinetic and isotherm models significantly explained the experimental data, and the observed correlations were unlikely to have occurred by chance.

In studies of reaction kinetics aimed at determining the time required for adsorption equilibrium to be reached under static conditions, the concentrations of individual reactants in solution were measured at intervals of 1 h, 3 h, 6 h, 10 h, 24 h, and 48 h. In addition, chemical equilibrium studies were conducted over a period of 48 h. Throughout these tests, the pH, temperature, and electrical conductivity were monitored using a portable SCHOTT meter (Mainz, Germany). All experiments were performed at room temperature, ranging from 20 to 22 °C. The concentrations of individual reactants were determined using the following analytical methods:

- PN-EN ISO 11885:2009: Heavy metals in solution were measured using ICP-ASA (atomic absorption spectrometry) and ICP-AES (inductively coupled plasma atomic emission spectrometry) from Thermo Scientific, Waltham, MA, USA [30];
- PN-ISO 9297:1994: Chloride ions were analyzed through titration (Mohr method) [31].

## 3. Results

### 3.1. Preliminary Flow Test Results

**Figure 3:**
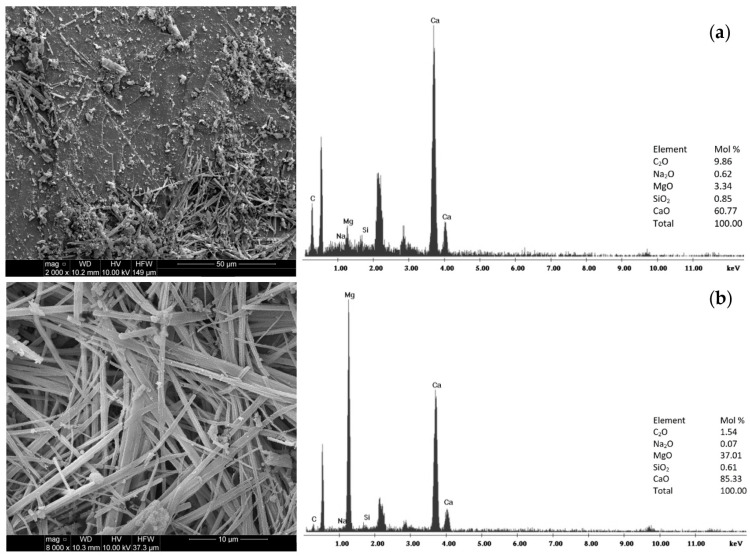
Microstructure and chemical composition analysis by SEM of (**a**) the surface of the MIX material and (**b**) MgO crystals on the surface of the MIX material.

The results of preliminary flow tests indicated that, during the contact between chloride ions and the materials present in the bed, the modified materials demonstrated a greater retention of chloride ions compared to their unmodified counterparts (refer to Figure 3). By analyzing the changes in the masses of the materials, reactor, and receiver before and after the test, we determined the degree of chloride ion reduction during contact with the tested materials, expressed as the removal ratio (R_R_%). Among the materials evaluated, MAC exhibited the highest removal ratio, with R_R_ = 60.07%, while MCA retained chloride ions at (R_R_ = 20.43%). In contrast, the unmodified materials displayed minimal reduction in chloride ions, with R_R_ = 0.57% for AC and R_R_ = 0.34% for CA.

No clogging of the material bed was observed during the experiment. The flow time of the solution through the beds of both modified and unmodified CA was, comparably, approximately 40 min. However, the modified AC bed exhibited a significantly longer flow time of approximately 70 min compared to the pure material bed, which registered about 50 min. Furthermore, the highest flow velocity of chloride ions through the bed-filled reactor was found in non-modified MCA (1.56 × 10^−5^ m/s and 1.54 × 10^−5^ m/s), whereas the lowest flow velocity was associated with MAC (9.50 × 10^−6^ m/s).

Based on the results obtained, it can be concluded that MAC is the most effective material for contaminant retention, while CA and MCA provide superior bed permeability. In order to enhance contaminant retention while reducing the risk of bed aggregation, a decision was made to modify the mixture of AC and CA with a 10% MgO solution.

### 3.2. Modification Results

The modification was executed at a weight ratio of AC to CA of 1:5. The resulting product will henceforth be referred to as a “MIX” throughout the paper. This selection was also driven by economic considerations, as utilizing a smaller quantity of the more expensive reactive material, activated carbon, for the composite is beneficial. Additionally, the incorporation of MgO is deemed necessary due to the low specific surface area of CA.

**Figure 4:**
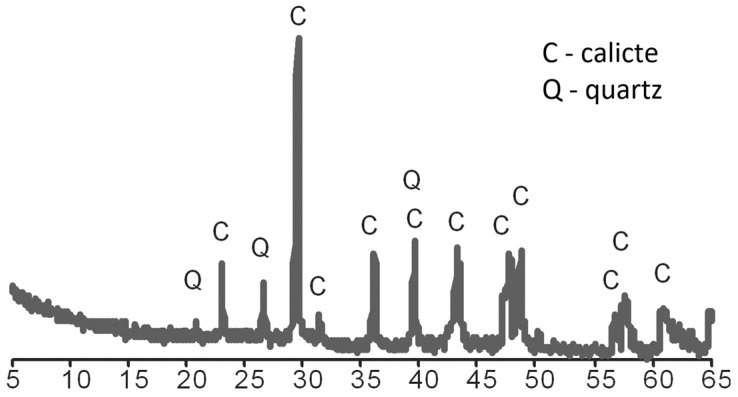
Diffractogram of MIX.

Figure 3 presents images of the composite’s microstructure and chemical composition, analyzed via scanning electron microscopy (FEG Quanta 250, Hillsboro, Oregon, USA) equipped with an EDS composition analysis system from EDAX. Figure 4 displays spectra of the composite obtained through a Philips X’pert APD X-ray diffractometer (Almelo, The Netherlands). The SEM analysis indicated that the test material is of a carbonate nature, which can be attributed to the higher proportion of CA in the sample. Chemical analyses revealed that the micro-area predominantly consisted of calcium, carbon, and oxygen (see Figure 3).

Moreover, numerous needle-like clusters of MgO crystals were observed on the surface of the sample, composed of magnesium, calcium, and oxygen. A diffraction analysis, analogous to that conducted for CA, confirmed that the principal mineral component is calcite (C), with interplanar distances of dhkl measuring 3.862, 3.040, 2.849, 2.494, 2.283, and 1.912 Å, alongside trace amounts of quartz (Q) characterized by dhkl values of 4.255, 3.344, and 2.283 Å (refer to Figure 4).

**Figure 5:**
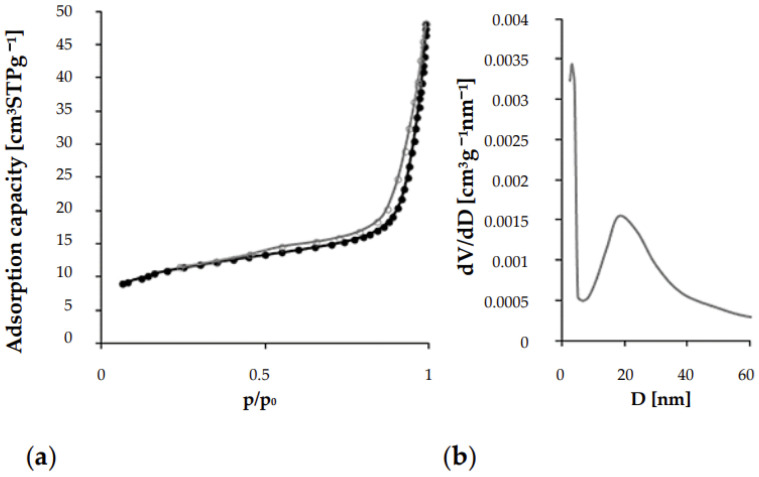
Plots showing (**a**) the nitrogen adsorption/desorption isotherms and (**b**) the pore volume distribution versus diameter (dV/dD) for the MIX sample.

In terms of specific surface area, the resultant material is classified as mesoporous, exhibiting a moderately developed specific surface area of approximately 38.73 m^2^/g (see Table 2). The structural analysis of the material reveals narrow pores in the form of tubules or fissures, as evidenced by the narrow desorption hysteresis loop illustrated in Figure 5. The pore volume distribution in relation to the diameter suggests variability in pore sizes. The average pore diameter is approximately 7 nm, while the graph presented in Figure 5 indicates a peak around 18 nm, suggesting a significant quantity of pores of that size. Furthermore, the height of this peak and its irregular shape further confirm the material’s heterogeneity regarding pore diameter.

### 3.3. Batch Test Results

**Figure 6:**
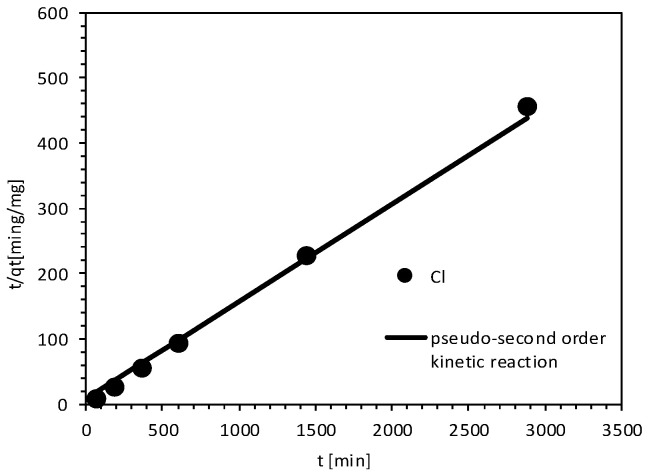
Pseudo-second-order reaction kinetics of Cl on MIX.

The results of the reaction kinetics for chloride ions during contact with MIX fitted the pseudo-second-order model very well. The values of the determination coefficients for Cl for MIX were 0.99. Figure 6 shows graphs of the reaction kinetics. The parameters and conditions determined from the kinetic studies are presented in Table 3.

**Figure 7:**
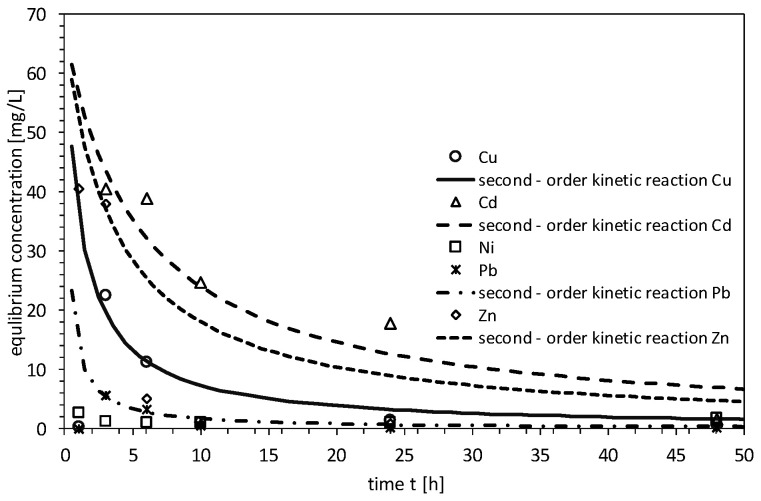
Second-order reaction kinetics of heavy metals on MIX.

**Figure 8:**
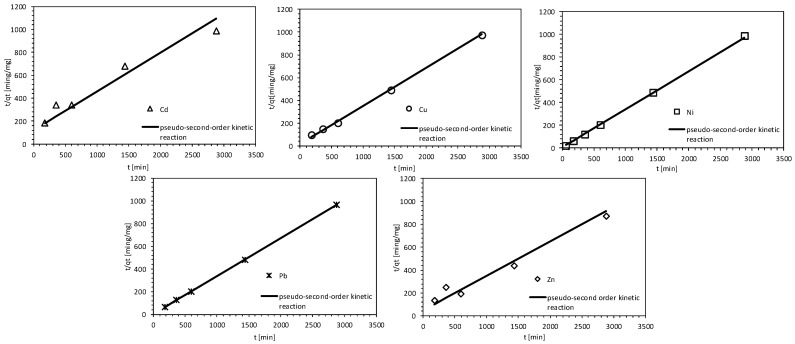
Pseudo-second order reaction kinetics of heavy metals on MIX.

In the study of the kinetics of reactions involving heavy metals, the best fit for the data was found using both the second-order and pseudo-second-order models. Figure 7 and Figure 8 provide a graphical interpretation of the average test results. For the second-order model, reaction equilibrium for cadmium, copper, zinc, and lead ions was reached after 48 h. However, the testing period was insufficient for nickel ions. The results indicated that copper (Cu), cadmium (Cd), and lead (Pb) ions were removed from the solution to the greatest extent, which was corroborated by the determination coefficients (R^2^) obtained: Cu—0.83, Cd—0.88, Pb—0.92.

The pseudo-second-order reaction kinetics model most accurately described the retention results for all ions present in the solution, particularly for copper (Cu), nickel (Ni), and lead (Pb), which had determination coefficients of 0.99. The coefficients for the other metals were as follows: Cd—0.93 and Zn—0.96. In addition, during the three-step diffusion–adsorption process, only cadmium ions were retained as contaminants, and their removal was proportional to t_1/2_. The model fit was confirmed by a coefficient of determination of 0.92 (see Table 3).

The Fisher test confirmed that all analyzed parameters (Cd, Cu, Pb, Zn, Cl, and Ni) followed the pseudo-second-order kinetic model with strong statistical significance (*p* < 0.05). The R^2^ values ranged from 0.75 to 0.99, indicating good-to-excellent model performance. Notably, both Pb (R^2^ = 0.92, F = 54.17) and Cd (R^2^ = 0.92, F = 72.35) exhibited particularly strong fits. Additionally, the extremely high F-values for Cl, Cu, Ni, and Pb (exceeding 2900) further support the reliability of the models, confirming that they effectively describe the adsorption kinetics and intraparticle diffusion behavior.

**Figure 9:**
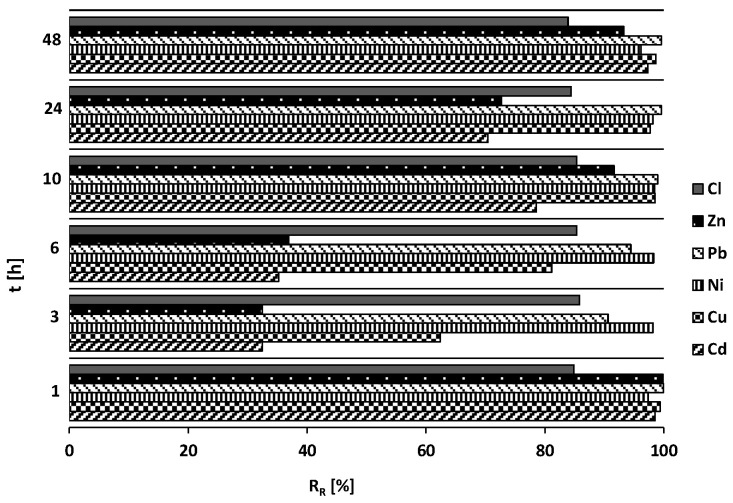
Removal of Cd, Cu, Ni, Pb, Zn, and Cl during contact with MIX.

**Figure 10:**
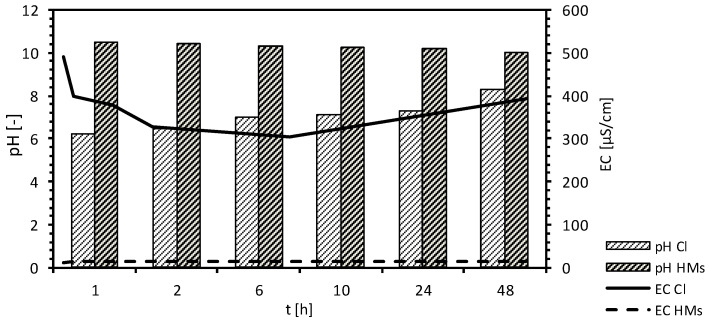
Conditions of kinetics studies on MIX for chloride (Cl) and heavy metals (HMs).

The removal expressed by R_R_ of the mixture of contaminants on the MIX is shown in Figure 9. The course of the graph demonstrated that nickel (98%), lead (98%), and copper (90%) ions were removed from the model solution to the highest extent. In contrast, chloride (Cl), zinc (Zn), and cadmium (Cd) ions were retained by the MIX to a lesser degree, with retention factors of 85%, 71%, and 69%, respectively. The chloride and lead ion retention process on the MIX was with the same intensity during the entire duration of the experiment. Figure 10 shows the changes in pH and electric conductivity (EC) during the kinetic studies.

During the kinetic studies, the pH of the chloride ion-contaminated solutions exhibited a consistent trend, remaining nearly stable throughout the observation period. Notable differences were recorded at the 1 h and 3 h marks, with the pH increasing from an initial value of 10.00 to 10.51 and 10.41, respectively. After 48 h, the pH was measured at 10.01. In contrast, the electrical conductivity (EC) values demonstrated an upward trend, increasing from an initial reading of 166 µS/cm to 491 µS/cm after 1 h, and subsequently to 400 µS/cm after 3 h. At the conclusion of the test, an EC measurement of 394 µS/cm was documented. In the case of the heavy-metal mixture solution, the pH increased from an initial value of 6.17 to 7.11 after 6 h, and further to 8.30 after 48 h. Conversely, the EC value displayed a slight decrease from the initial measurement of 17.7 µS/cm, declining to 15.08 µS/cm at the 48 h mark.

**Figure 11:**
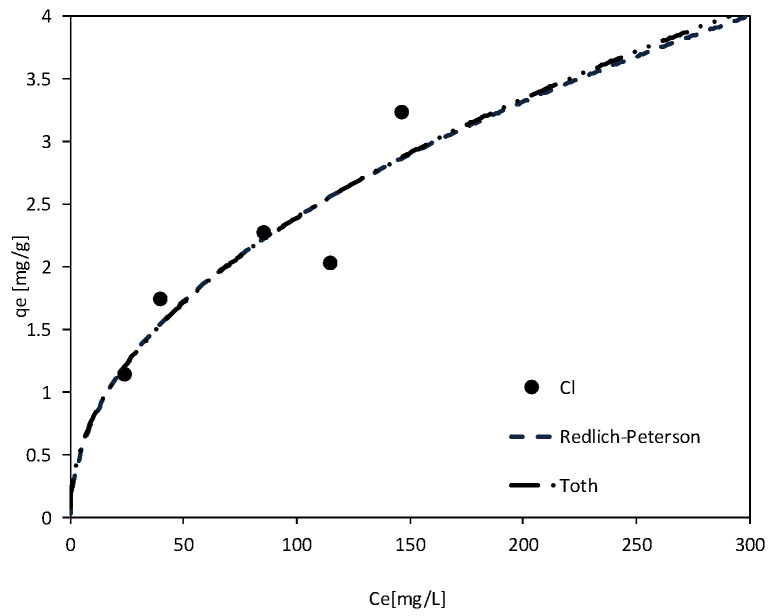
Adsorption model isotherms describing Cl chemical equilibrium reactions during contact with MIX.

In a series of chemical equilibrium studies conducted on modified materials and an impurity mixture solution, the Toth and Redlich–Peterson models emerged as the most accurate representations for chloride ions (Cl^−^). The determination coefficients for these models were recorded as R^2^ = 0.80 for the Toth model and R^2^ = 0.79 for the Redlich–Peterson model. The adsorption isotherms for the modified composite (MIX) are presented in Figure 11.

**Figure 12:**
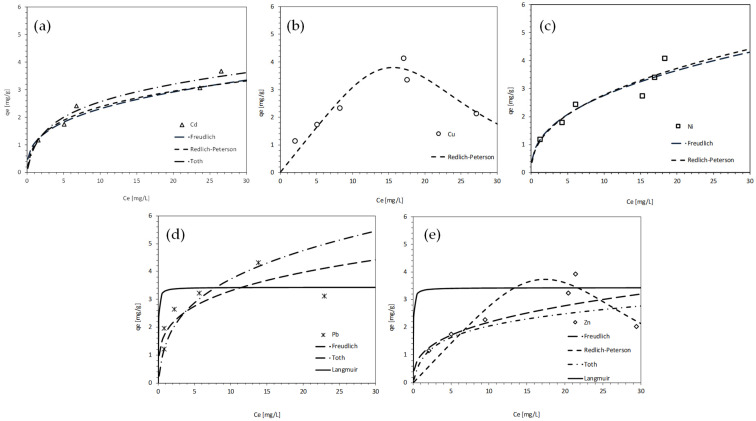
Adsorption model isotherms describing heavy metals: (**a**) Cd, (**b**) Cu, (**c**) Ni, (**d**) Pb, and (**e**) Zn chemical equilibrium reactions during contact with MIX.

**Figure 13:**
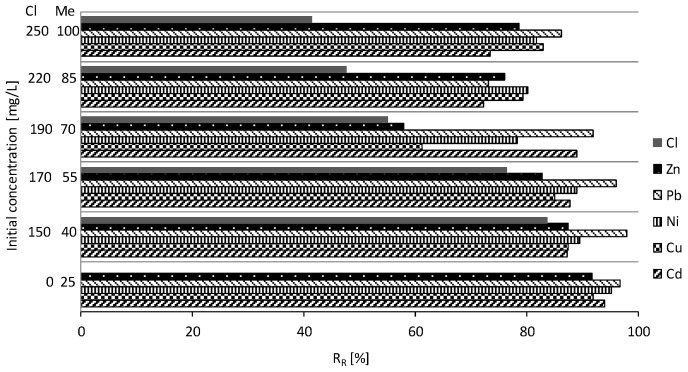
Removal of Cd, Cu, Ni, Pb, Zn, and Cl during contact with MIX.

In further studies utilizing MIX from the heavy metal model solution, monolayer adsorption was found to be one of the predominant mechanisms for the removal of most heavy metal ions, as evidenced by the correlation at a satisfactory level with the Langmuir isotherm model (R^2^ > 0.80 for Cu, Ni, Pb, Zn). The low R_L_ coefficient values for these metals (ranging from 0.0008 to 0.018) suggest a high degree of adsorption onto the composite. Additionally, the adsorption data were well-aligned with the Freundlich isotherm, suggesting a heterogeneous, microporous surface, with cadmium (Cd) and nickel (Ni) both demonstrating high determination coefficients (R^2^ = 0.97). However, the Redlich–Peterson model offered the most comprehensive description by integrating both physical and chemical interactions, effectively bridging the Langmuir and Freundlich behaviors. This model provided excellent fits for cadmium (Cd), copper (Cu), and nickel (Ni), with an R^2^ of 0.96, and a satisfactory fit for zinc (Zn), with an R^2^ of 0.91. Fisher’s F-test confirmed the statistical validity of all isotherm models (*p* ≤ 0.05). In the Langmuir model, copper (Cu), nickel (Ni), lead (Pb), and zinc (Zn) exhibited strong fits, particularly lead (Pb), which had an exceptionally high R^2^ and a low *p*-value. The Freundlich model was significant only for cadmium (Cd), nickel (Ni), and lead (Pb). All parameters of the Redlich–Peterson model were significant (R^2^ ≥ 0.91), although chloride (Cl) demonstrated a weaker fit with an R^2^ of 0.79. The Toth model also met the significance criteria (*p* ≤ 0.05) for all analytes. Chloride (Cl) and cadmium (Cd) fit well (R^2^ ≥ 0.75), while lead (Pb) and zinc (Zn) displayed moderate fits. The equilibrium results are illustrated in Figure 12 and Figure 13 and presented in Table 4.

Figure 13 illustrates the reduction of chloride ions from a series of chemical reaction equilibrium tests for MIX, represented by the removal ratio (R_R_) as a function of the initial concentration of the contaminant in the model solution. The removal ratio of chloride and heavy metal ions during contact with the modified material was determined using Equation (1). The removal ratio (R_R_) exhibited an increase corresponding to higher initial concentrations of chloride ions in the solution. On average, the modification of reactive materials elevated the RR value for MIX from 5% to 60%. As illustrated in the graph, the metals that were most effectively removed from the solution were lead, copper, and zinc, in that order. In contrast, other ions demonstrated a propensity for retention at lower initial concentrations. The average retention rates for the individual metals were as follows: Pb: 90%; Cd: 86%; Ni: 85%; Cu: 81%; and Zn: 79%.

During the equilibrium tests, the pH of the solutions contaminated with chloride ions remained relatively stable at 10.00 ± 0.60. The same consistency was observed in the electrical conductivity (EC) values across the individual solutions. In the initial solutions with the lowest concentrations of chloride ions, the EC values increased from 192 μS/cm to 1164 μS/cm. In samples with the highest chloride ion concentrations, the EC values rose from 323 μS/cm to 1242 μS/cm. In the case of the heavy-metal mixture solution, the pH increased from an initial value of 6.30–8.23 to 11.00 ± 0.70. In contrast, the EC value showed only slight variations from the initial measurement, with deviations of ±0.2 μS/cm. For samples with low concentrations of heavy metals, the EC was measured at 6.68 μS/cm, whereas in samples with higher concentrations, the EC was recorded at 25.4 μS/cm.

## 4. Discussion

The results of this study provide strong evidence for the effectiveness of a composite material (MIX), which is made of modified construction aggregate and activated carbon, in removing chloride ions and heavy metal contaminants from groundwater. The study supports the hypothesis that surface modification—especially with the addition of magnesium oxide (MgO)—significantly enhances the sorptive and reactive properties of the composite.

The modified MIX achieved a notable chloride ion removal efficiency of up to 76% at an initial concentration of 170 mg/L. This indicates its promising capacity for the treatment of saline or chloride-contaminated water, particularly in areas affected by road salt runoff or seawater intrusion. The application of the Toth and Redlich–Peterson isotherms suggests that chloride adsorption was primarily driven by heterogeneous surface interactions. The observed variations in adsorption energies likely correspond to the microstructural changes introduced by MgO crystallization, which creates a distribution of active sites with differing affinities.

These findings are consistent with previous studies that highlight the importance of surface heterogeneity in chloride adsorption [32]. They further extend our understanding by demonstrating that surface-engineered materials derived from industrial byproducts can serve as cost-effective and environmentally sustainable alternatives to traditional adsorbents.

In terms of heavy metal removal, the composite demonstrated high efficiency across various metal ions, including Pb^2+^, Cd^2+^, and Zn^2+^. The removal mechanism was found to be dual: adsorption onto the MIX surface and chemical precipitation as hydroxides, which were facilitated by elevated solution pH values (9–11). The basic pH environment arose from the mineral composition of the road aggregate and the presence of MgO, which promoted the formation of magnesium hydroxide species. These conditions favor the precipitation of metal hydroxides, effectively reducing dissolved metal concentrations, as supported by similar findings in Navarro, Cardell, and Martín [33]. It is essential to emphasize that, although kinetic and equilibrium models were utilized under the assumption that adsorption serves as the primary mechanism for removal, the possible impact of hydroxide precipitation may alter the interpretation of concentration changes. This consideration represents a limitation that has been acknowledged in this study.

For heavy metals, the Redlich–Peterson model provided the best overall fit for Cd, Cu, Ni, and Zn ions (R^2^ = 0.96–0.91), indicating mixed-mode sorption behavior involving both physisorption and chemisorption, as well as surface heterogeneity. The Langmuir model showed a suitable correlation with removal data (R^2^ > 0.80 for Cu, Ni, Pb, and Zn), suggesting that monolayer adsorption was a dominant mechanism. The Langmuir model displayed particularly strong correlations (R^2^ > 0.8). The dimensionless separation factor (RL), ranging from 0.0008 to 0.01, indicated highly favorable adsorption conditions. The low RL values (0.0008–0.018) further imply efficient adsorption. Meanwhile, high R^2^ values for the Freundlich model (e.g., 0.97 for Cd and Ni) reinforced this interpretation, indicating multilayer adsorption on a heterogeneous surface, consistent with the morphology of MgO-coated materials [32,33]. This aligns with the literature on activated carbon composites [34,35], reinforcing the validity of the MIX formulation as a competitive alternative to more costly treatment media.

Compared to prior research, this study offers new insights by combining modified construction waste materials with activated carbon and MgO to create a multifunctional adsorbent. Previous studies have examined MgO for heavy metal removal [36] and activated carbon for organic and ion adsorption [37], but this study is one of the first to integrate these approaches into a low-cost, scalable composite.

From a practical perspective, using reclaimed road aggregate not only reduces material costs but also aligns with circular economy principles in environmental engineering. The broad pH tolerance and dual removal mechanisms enhance the versatility of the MIX across various groundwater chemistries, especially in decentralized or emergency water treatment systems.

While the findings are encouraging, several areas require further investigation. First, the long-term stability of the composite under field conditions, including the potential leaching of retained contaminants, needs to be evaluated. Second, the performance of MIX in complex, real-world groundwater matrices containing competing ions and natural organic matter remains unexamined. Additionally, future studies should optimize the MgO dosage to balance the removal efficiency with the material costs and environmental impacts.

Furthermore, kinetic studies and column experiments will be essential to assess the scalability and dynamic behavior of MIX in continuous-flow systems. Exploring regeneration and reuse cycles of the composite material will also be critical for its practical deployment.

## 5. Conclusions

The experimental results confirm the effectiveness of the composite material (MIX), consisting of modified construction aggregate, activated carbon, and magnesium oxide (MgO), in removing chloride ions and heavy metals from contaminated groundwater. The adsorption kinetics for these contaminants align with the pseudo-second-order model, indicating that chemisorption is the primary mechanism. Coefficients of determination (R^2^) for Cu^2+^, Ni^2+^, Pb^2+^, Cd^2+^, and Zn^2+^ range from 0.93 to 0.99, highlighting strong chemical adsorption.

Equilibrium for Cd^2+^, Cu^2+^, Zn^2+^, and Pb^2+^ was reached within 48 h, while Ni^2+^ showed slower kinetics. The intraparticle diffusion model confirmed that intraparticle diffusion plays a significant role in cadmium removal (R^2^ = 0.92). The removal efficiencies were highest for Ni (98%), Pb (98%), and Cu (90%), with lower rates for Cl^−^ (85%), Zn (71%), and Cd (69%), suggesting more efficient metal ion adsorption.

The equilibrium data were best described by the Langmuir and Redlich–Peterson models, indicating favorable monolayer adsorption, while the Freundlich model was effective for Cd and Ni (R^2^ = 0.97). Surface modification increased the removal ratios by up to 60%, underscoring the roles of MgO and activated carbon in enhancing the adsorption potential. These findings support the use of MIX in decentralized water treatment, offering high efficiency, fast kinetics, and low-cost materials. The increased removal ratios due to surface modification (up to a 60% improvement) emphasize the critical role of MgO and activated carbon in enhancing the surface reactivity and adsorption potential of the base material. These findings align with prior research on the MgO-based sorbents and modified carbons used for the removal of heavy metals and ions from aqueous systems. From an application perspective, the composite material presents significant advantages: high removal efficiency across various contaminants, relatively fast adsorption kinetics, and the use of low-cost, recycled construction waste. These attributes make the MIX composite a promising candidate for decentralized water treatment technologies, especially in contexts with complex contaminant mixtures. However, future research should focus on quantitatively distinguishing between the adsorption and precipitation mechanisms involved in metal removal. This differentiation can be accomplished through the application of advanced speciation analyses and complementary experimental methodologies. Moreover, an analysis applying multi-component equilibrium models that account for competitive adsorption behavior will be performed.

## Figures and Tables

**Table 1:** Preliminary flow test results.

Materials	CA		MCA		AC		MAC	
Conditions	Before	After	Before	Before	After	After	Before	After
volume of solution in syringe [mL]	20.0	9.5	20.0	9.5	20.0	9.5	20.0	9.5
mass of material [g]	6.7000	6.7042	5.0935	5.5171	1.6936	1.7179	1.9689	9.0299
mass of quartz wool [g]	0.2080	0.5198	0.208	0.5033	0.2080	0.6185	0.208	0.4613
mass of reactor [g]	66.9382	70.7000	65.28	68.7893	65.9535	69.781	62.1948	66.6346
mass of vessels [g]	55.0165	55.9210	39.4487	40.8032	50.3204	51.1857	55.5052	56.115
volume of r-liquid in receiver [mL]		5.5		5.5		5.5		5.5
velocity of flow through bed [m/s]	1.56 × 10^−5^		1.54 × 10^−5^		1.39 × 10^−5^		9.50 × 10^−6^	
t [s]	2491.8		2593.2		2902.2		4206.0	
R_R_ [%]	0.34		20.43		0.57		60.07	

**Table 2:** Results of determining the specific surface area of materials by nitrogen adsorption/desorption.

Material	S_BET_ [m^2^/g]	V_mic_ [cm^3^/g]	S_mic_ [m^2^/g]	V_mes_ [cm^3^/g]		S_mes_ [m^2^/g]		D [nm]		D_BJH_/D_BJH_ [nm]
				Ads.	Des.	Ads.	Des.	Ads.	Des	
**AC**	856.05	0.1600	366.41	0.51	0.50	177.10	92.31	3.65	4.85	0.40
**CA**	2.82	0.0002	0.28	0.00	0.00	0.95	1.35	12.12	11.65	14.13
**MIX**	38.73	0.0700	-	0.07	0.07	22.74	17.99	12.02	13.61	6.91

Where S_BET_ [m^2^/g]—specific surface area calculated using the BET (Brunauer–Emmett–Teller) method. V_mic_ [cm^3^/g]—micropore volume. S_mic_ [m^2^/g]—micropore surface area. V_mes_ [cm^3^/g]—mesopore volume. S_mes_ [m^2^/g]—mesopore surface area. D [nm]—average pore diameter. D_BJH_/D_BJH_ [nm]—pore diameter calculated using the BJH (Barrett–Joyner–Halenda) method.

**Table 3:** Parameters and conditions determined from kinetic studies.

	Contamination	Cl	Cd	Cu	Ni	Pb	Zn
Model							
Second- order kinetic	Cs [mg/L]	-	1.6	0.78	-	0.23	0.95
	k_2_ [min^−1^]	-	0.0027	0.0121	-	0.0559	0.004
	R^2^ [-]	-	0.88	0.83	-	0.92	0.75
	F [-]	-	29.31	32.10	-	54.17	15.19
	*p*-value [-]	-	0.012	0.011	-	0.005	0.029
Pseudo- second-order kinetic	Cs [mg/L]	13.72	1.6	0.78	1.76	0.23	0.95
	k_p2_ [min^−1^]	0.098	0.001	0.007	0.017	0.037	0.002
	R^2^ [-]	0.99	0.93	0.99	0.99	0.99	0.96
	F [-]	95,679.11	93.91	2900.70	9768.36	91,246.94	121.73
	*p*-value [-]	7.45 × 10^−8^	0.002	7.45 × 10^−8^	6.29 × 10^−10^	8.00 × 10^−8^	0.002
Intraparticle diffusion	Cs [mg/L]	-	1.6	-	-	-	-
	k_int_ [min^−1^]	-	0.0574	-	-	-	-
	R^2^ [-]	-	0.92	-	-	-	-
	F [-]	-	72.35	-	-	-	-
	*p*-value [-]	-	0.003	-	-	-	-
pH [-]	10.00–10.41			6.17–8.30			
EC [µS/cm]	176.00–491.00			17.70–15.08			

Where Cs [mg/L]—equilibrium concentration of adsorbate on the adsorbent; k_2_ [min^−1^]—rate constant of the second-order kinetic model; k_p2_ [min^−1^]—rate constant for pseudo-second-order kinetics model; k_int_ [min^−1^]—rate constant for the intraparticle diffusion model; R^2^ [-] coefficient of determination; F [-]—F-value from Fisher’s test; measures the overall significance of the regression model; *p*-value (F significance) [-]—significance level from statistical testing; determines whether the regression equation explains a significant portion of the variability in the dependent variable.

**Table 4:** Parameters and conditions determined from chemical equilibrium studies.

	Contamination	Cl	Cd	Cu	Ni	Pb	Zn
Model							
**Langmuir**	q_max_ [-]	-	-	2.69	4.38	3.43	2.66
	K_L_ [L/mg]	-	-	10.94	3.92	25.97	7.44
	R_L_ [L/mg]	-	-	0.002	0.005	0.001	0.003
	R^2^ [-]	-	-	0.80	0.88	0.96	0.79
	F [-]	-	-	16.12	53.68	101.62	14.68
	*p*-value [-]	-	-	0.02	0.005	0.001	0.018
**Freundlich**	K_F_ [mg/L]	-	1.02	0.98	1.06	1.79	0.98
	N_F_ [-]	-	0.38	0.38	0.44	0.26	0.38
	R^2^ [-]	-	0.95	0.62	0.97	0.71	0.65
	F [-]	-	65.96	6.67	113.94	9.61	7.44
	*p*-value [-]	-	0.004	0.061	0.002	0.036	0.053
**Redlich–Peterson**	K_R_ [L/mg]	0.62	2.43	0.32	89.56	-	0.29
	B_R_ [-]	1.97	1.64	0.00	85.09	-	0.00
	β [-]	0.55	0.73	3.99	0.56	-	4.05
	R^2^ [-]	0.79	0.96	0.96	0.96	-	0.91
	F [-]	12.21	52.69	47.81	78.23	-	27.64
	*p*-value [-]	0.040	0.005	0.002	0.003	-	0.006
**Toth**	K_T_ [-]	0.26	1.43	-	-	1.82	1.27
	b_T_ [-]	0.001	0.831	-	-	0.54	0.911
	β [-]	1.93	1.39	-	-	1.48	1.31
	R^2^ [-]	0.80	0.75	-	-	0.66	0.65
	F [-]	12.30	33.57	-	-	19.05	8.11
	*p*-value [-]	0.039	0.010	-	-	0.012	0.046
**pH [-]**	10.00–10.30			6.28–11.79			
**EC [µS/cm]**	190.00–1331.00			7.68–25.50			

Where q_max_ [-]—maximum adsorption capacity; K_L_ [L/mg]—Langmuir equilibrium constant; R_L_ [L/mg]—separation factor (dimensionless); K_F_ [mg/L]—Freundlich constant; N_F_ [-]—the Freundlich exponent/heterogeneity factor; K_R_ [L/mg]—Redlich–Peterson isotherm constants; B_R_ [-]—Redlich–Peterson isotherm constants; β [-]—Redlich–Peterson exponent; K_T_ [-]—Toth isotherm constant; b_T_ [-]—Toth model constant; β [-]—Toth exponent/heterogeneity parameter; R^2^ [-] coefficient of determination; F [-]—F-value from Fisher’s test; measures the overall significance of the regression model; *p*-value (F significance) [-]—significance level from statistical testing; determines whether the regression equation explains a significant portion of the variability in the dependent variable.

## Data Availability

The original contributions presented in this study are included in the article. Further inquiries can be directed to the corresponding author.
